# Temporal transcriptomic profiling elucidates sorghum defense mechanisms against sugarcane aphids

**DOI:** 10.1186/s12864-023-09529-5

**Published:** 2023-08-05

**Authors:** Heena Puri, Sajjan Grover, Lise Pingault, Scott E. Sattler, Joe Louis

**Affiliations:** 1 Department of Entomology, University of Nebraska-Lincoln, Lincoln, NE 68583 USA; 2 Wheat, Sorghum, and Forage Research Unit, U.S. Department of Agriculture-Agricultural Research Service, Lincoln, NE 68583 USA; 3 Department of Biochemistry, University of Nebraska-Lincoln, Lincoln, NE 68583 USA

**Keywords:** Differentially expressed genes, RNA-sequencing, Plant defenses, Sorghum, Sugarcane aphid

## Abstract

**Background:**

The sugarcane aphid (SCA; *Melanaphis sacchari*) has emerged as a key pest on sorghum in the United States that feeds from the phloem tissue, drains nutrients, and inflicts physical damage to plants. Previously, it has been shown that SCA reproduction was low and high on sorghum SC265 and SC1345 plants, respectively, compared to RTx430, an elite sorghum male parental line (reference line). In this study, we focused on identifying the defense-related genes that confer resistance to SCA at early and late time points in sorghum plants with varied levels of SCA resistance.

**Results:**

We used RNA-sequencing approach to identify the global transcriptomic responses to aphid infestation on RTx430, SC265, and SC1345 plants at early time points 6, 24, and 48 h post infestation (hpi) and after extended period of SCA feeding for 7 days. Aphid feeding on the SCA-resistant line upregulated the expression of 3827 and 2076 genes at early and late time points, respectively, which was relatively higher compared to RTx430 and SC1345 plants. Co-expression network analysis revealed that aphid infestation modulates sorghum defenses by regulating genes corresponding to phenylpropanoid metabolic pathways, secondary metabolic process, oxidoreductase activity, phytohormones, sugar metabolism and cell wall-related genes. There were 187 genes that were highly expressed during the early time of aphid infestation in the SCA-resistant line, including genes encoding leucine-rich repeat (LRR) proteins, ethylene response factors, cell wall-related, pathogenesis-related proteins, and disease resistance-responsive dirigent-like proteins. At 7 days post infestation (dpi), 173 genes had elevated expression levels in the SCA-resistant line and were involved in sucrose metabolism, callose formation, phospholipid metabolism, and proteinase inhibitors.

**Conclusions:**

In summary, our results indicate that the SCA-resistant line is better adapted to activate early defense signaling mechanisms in response to SCA infestation because of the rapid activation of the defense mechanisms by regulating genes involved in monolignol biosynthesis pathway, oxidoreductase activity, biosynthesis of phytohormones, and cell wall composition. This study offers further insights to better understand sorghum defenses against aphid herbivory.

**Supplementary Information:**

The online version contains supplementary material available at 10.1186/s12864-023-09529-5.

## Background

Sorghum (*Sorghum bicolor*) is an important cereal crop grown worldwide and specifically in the United States, it was planted on 2.06 million hectares with an annual production of 9.47 million metric tons in 2021 [1]. In dry climatic regions of the world, sorghum grains are used as food source because of its high drought tolerance [2]. In the United States, it is mainly used for livestock feed and biofuel production, and currently it is also gaining the attention of the food industry due to its high nutritional value [3]. However, the outbreak of sugarcane aphid (SCA; *Melanaphis sacchari*) in 2013 in the United States has impacted the sorghum yield causing more than 50% losses in the subsequent years [4]. SCA was first reported on sorghum in Texas Gulf Coast and Louisiana [4]. SCA moves with wind currents and has invaded 20 states since 2013 threatening sorghum production in the United States [5]. SCA feeds on the lower side of leaves by inserting its piercing-sucking mouthparts, known as stylets, into the phloem cells thereby consuming nutrients present in the plant sap. Drainage of nutrients from plants after SCA feeding also causes the leaves to turn yellowish, purple, and ultimately brown upon the death of leaf tissue. Complete coverage of plants with aphids can cause plant stunting followed by death [6]. Aphids also secrete sugary substance known as honeydew, which allows the growth of sooty mold and results in reduced leaf area for light absorption and photosynthesis in plants, thus declining the photosynthetic efficiency of infested plants. The high dispersal ability and reproductive capability allow SCA to swiftly achieve high population densities and cause severe yield losses [7].

Host plant resistance has proven to be highly economical and eco-friendly long term insect management strategy [8] that has been broadly used for the management of other sorghum pests such as greenbugs (*Schizaphis graminum*) and corn leaf aphids (*Rhopalosiphum maidis*) [9, 10]. In last few years, numerous attempts were made to identify different sources of aphid resistance in sorghum [11–13]. Sorghum genotypes that have displayed resistance to greenbugs have also exhibited resistance to SCA [11, 13–15], which offer greater potential for deploying the resistant cultivars in sorghum breeding programs. The resistant plant varieties can defend themselves by displaying (i) antibiosis, (ii) antixenosis, or (iii) tolerance [16–18]. Antibiosis occurs when resistant plants impose adverse effects on the insect growth, survival, and fecundity. Antixenosis, also known as non-preference, is where plants display non-preferred characters, such as the presence of surface trichomes, thorns, spines and hair on leaves, and stems, which are unattractive to the incoming insects thereby affecting the behavior of the insects [17]. Tolerance is the most durable category of resistance, which is the ability of the plant to grow and sustain under insect attack without much compromising the crop yield traits as compared to the susceptible plants. Moreover, tolerant plants do not impose negative impact on insect biology that delays the development of insect biotypes [19].

Plants utilize myriad of defense strategies to counteract the insect attack [20] and those strategies may also depend on the mode of insect feeding. Aphid saliva is a powerful component involved in mediating plant defenses, which can either elicit or suppress the defenses [21]. When aphids begin feeding on plants, it releases two types of saliva: i) gelling saliva that acts as a stylet sheath to protect the stylet from injury during penetration into the plant tissue and ii) watery saliva that helps in ingestion of plant sap [22]. Aphid watery saliva also contains salivary proteins, peroxidases, pectinases, carbohydrates, phospholipids, and enzymes that can modulate plant defenses [23]. Additionally, aphids release effector molecules into the plants through its saliva, which when recognized by R-proteins can trigger immunity in plants [24]. Cysteine-protease cathepsin B3, a potential elicitor present in green peach aphid (*Myzus persicae*) salivary glands initiated the plant defenses by binding to ENHANCED DISEASE RESISTANCE 1-like (EDR1-like) kinase in tobacco plant and leading to oxidative burst [25]. The phytohormones, for example, jasmonic acid (JA), salicylic acid (SA), ethylene, cytokinins, and abscisic acid play a significant role in modulating plant defenses against herbivory. Additionally, aphid feeding also alters genes regulating plant defenses such as pathogen-related (PR) proteins, defense signaling and metabolic pathways [26]. For example, potato aphid (*Macrosiphum euphorbiae*) can stimulate the expression of PR proteins on tomato plants that is induced by SA [27]. Similarly, in Arabidopsis, green peach aphid feeding induces the expression of defense genes *β-1–3 glucanse* and *PR1*, and *Plant Defensin 1.2*¸which are markers for SA and JA/ethylene pathways, respectively [28]. Greenbugs feeding on sorghum induces the expression of SA-related *PR* genes such as *PR10*, thaumatin-like proteins, chitinases and glucanases [29]. In addition to SA-regulated defense, JA is also involved in mediating defenses against phloem-feeding insects. Exogenous application of methyl jasmonate on plants deters aphid from settling on sorghum [15, 29]. Furthermore, plants also produce different secondary metabolites, which can be constitutively present in plants called phytoanticipins like benzoxazinoids and glucosinolates or induced upon insect feeding known as phytoalexins such as alkaloids, isoflavonoids, and terpenoids [30]. These metabolites are known to provide antibiotic effects against insects [24] and impair their sustained feeding on plants.

Understanding the mechanism of resistance is important to fully exploit the available sources of genetic resistance and to enhance our knowledge of plant–insect interactions for the development of novel insect management strategies. Different sorghum-resistant hybrids against SCA have been developed and fewer studies have reported the possible molecular mechanisms of sorghum resistance to SCA [31–33]. Previously, it was documented that the transcriptional responses of sorghum to SCA attack were more evident in resistant line compared to susceptible plants and these responses were mostly associated with phytohormones signaling, glutathione biosynthesis, and secondary metabolites [31]. Similarly, plant resistance genes related to nucleotide-binding-site, leucine-rich repeat (NBS-LRR) were also reported to be involved in sorghum resistance against SCA [32]. Previously, *RMES1* (Resistance to *Melanaphis sacchari*) was identified and mapped as the dominant resistance gene on chromosome 6 exhibiting resistance to SCA [34]. WRKY transcription factor 86 (*SbWRKY86*) (*Sobic.009G238200*) has also been identified in sorghum as a candidate gene involved in providing defense against SCA [35]. Furthermore, *R*-genes (plant resistance genes) in sorghum, for example, receptor-like kinase (RLK) genes, NBS-LRR, and receptor-like protein (RLP) genes have altered expression levels in the resistant sorghum line upon aphid infestation that can be implied for providing resistance to greenbugs [36]. Genome-wide association mapping of sorghum has revealed defense related genes like LRR, flavonoid biosynthesis, 12-oxo-phytodienoic reductase, WRKY transcription factors, lipoxygenases, and Avr proteins can be potential sources for sorghum breeding for aphid resistance [37]. Additionally, comparison of sorghum responses at early and late time points after aphid infestation distinguished the plant responses over time [33]. Resistant sorghum line showed upregulation of genes related to protein and lipid binding and autophagy, transcription initiation at 4 to 15 days post infestation (dpi) and gene responses to external biotic stimuli and stress, cell communication and transferase activities increased from 4 to 12 dpi [33].

The plants rapidly activate their defense machinery in response to insect attack. Upon recognition of the insect herbivory, plants trigger the early signaling events such as Ca^2+^ signaling, production of reactive oxygen species (ROS) followed by induction of phytohormones, gene activation and changes at the metabolic levels [38]. The ability of plants to identify and respond to an attack determines its capabilities to defeat the insect attack. Therefore, the current study was focused to assess the temporal plant responses to SCA feeding, which included short-term and prolonged aphid feeding times on sorghum plants. We used the previously identified SCA-resistant (SC265) and susceptible (SC1345) sorghum lines [15, 19], which are part of the founder nested association mapping (NAM) population [39], to understand the sorghum defense mechanism(s) against SCA using a transcriptomic approach. The objectives of the study were to identify the differentially expressed genes (DEGs) in sorghum in response to SCA feeding and to describe their role in plant defenses.

## Results

### Sorghum transcriptomic response to SCA attack

**Fig. 1:**
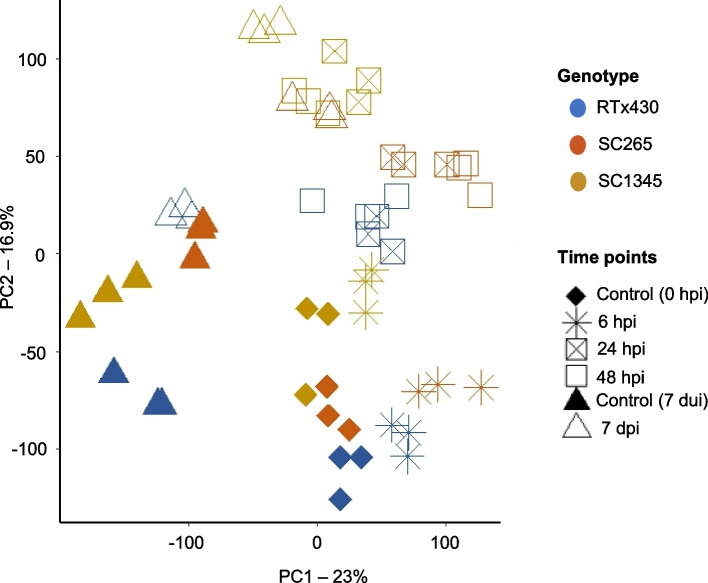
Principal component analysis of 26,794 genes expressed at least at one-time point. Colors represent the sorghum plants: RTx430, SCA-resistant (SC265), and SCA-susceptible (SC1345) sorghum lines. Shapes represent the time point of sample collection. Time points are grouped into early (0, 6, 24, and 48 hpi) and late (7 dui and 7 dpi) categories. Controls are at 0 h for the early time points and 7 dui for the late time point. hpi = hours post infestation, dui = days uninfested, and dpi = days post infestation

RNA-seq was deployed to determine the global transcriptional response to SCA infestation in reference (RTx430), SCA-resistant (SC265), and SCA-susceptible (SC1345) sorghum plants. We used principal component analysis (PCA) to determine the variation among lines at early and late time points after aphid infestation. PCA of 26,794 genes differentially expressed in at least one condition was done and PCA1 accounted for 23% of the variation, differentiating the transcriptome of early (i.e., 0, 6, 24, 48 hpi) and late (i.e., 7 day) time points corresponding to all sorghum lines (Fig. 1). PCA2 accounted for 16.9% of the variation; samples collected at 6 hpi were grouped with their control (0 h). PCA2 separated samples collected at 24 hpi/48 hpi/7 dpi from samples collected at 0 hpi/6 hpi/7 day uninfested control plants. These results show the high impact of SCA feeding after 24 hpi. SCA feeding at 6 hpi may not involve a large transcriptomic reorganization in sorghum plants (Fig. 1). The samples from SC265 and SC1345 were separated from RTx430 at 7 dpi but not in case of control samples collected at 7 days.

**Fig. 2:**
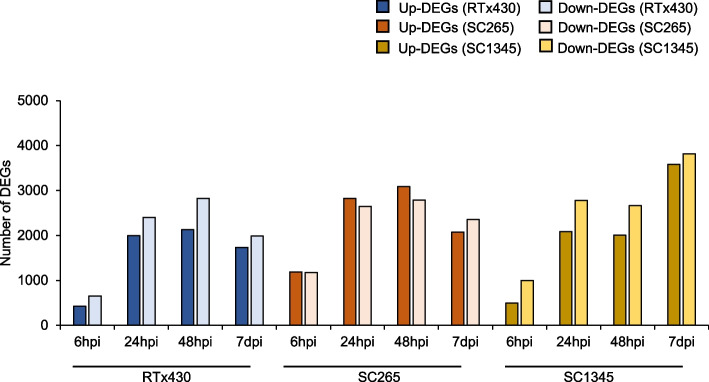
Total number of differentially expressed genes (DEGs). Number of upregulated and downregulated DEG in RTx430, SCA-resistant (SC265), and SCA-susceptible (SC1345) sorghum lines at different time points 6, 24, 48 hpi, and 7 dpi. hpi = hours post infestation and dpi = days post infestation

**Fig. 3:**
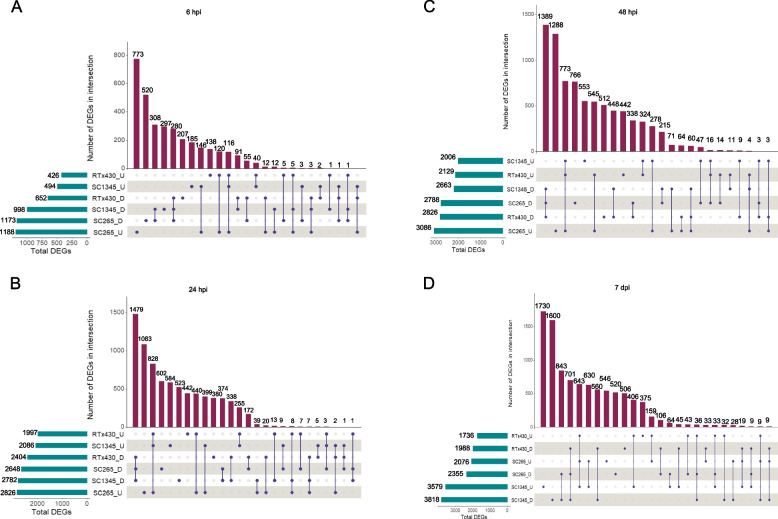
Upset intersection plots of the total number of differentially expressed genes (DEGs) [upregulated (U) and downregulated (D); *P* < 0.05, fold change > 2] present in a sorghum line at a given time point on horizontal bars (numbers shown on the left of each horizontal bar) and the total number of DEGs common in different sorghum lines represented by vertical bars (numbers shown at the top of each vertical bar). Vertical lines joining the points depicts that the sorghum line corresponding to the point have common DEGs in RTx430, SCA-resistant (SC265), and SCA-susceptible (SC1345) sorghum lines. **A** DEGs at 6 hpi, **B** DEGs at 24 hpi, **C** DEGs at 48 hpi, **D** DEGs at 7 dpi

In the SCA-resistant line (SC265), aphid herbivory resulted in increased number of upregulated genes compared to downregulated genes at early time points (Figs. 2 and 3A, B and C) and the number of DEGs downregulated was higher at 7 dpi (Figs. 2 and 3D). However, in RTx430 and SCA-susceptible (SC1345) plants, the number of upregulated DEGs were lower than the downregulated genes at all the time points (Figs. 2 and 3). As the aphid feeding progressed for 48 h, the total number of DEGs were increased in RTx430 and resistant line but prolonged feeding for 7 days led to sharp decrease in overall DEGs. In the SCA-susceptible line, aphid feeding led to a gradual increase in total number of DEGs until 7 dpi with a small drop in DEGs at 48 hpi (Figs. 2 and 3). Specifically, the total number of DEGs exclusively upregulated in RTx430 at 6, 24, and 48 hpi were 138, 442, and 442 respectively, and DEGs uniquely upregulated in SC265 at 6, 24, and 48 hpi were 773, 1083, and 1288, respectively (Fig. 3). However, at a later point (i.e., 7 dpi), the unique DEGs upregulated in RTx430 and SC265 plants decreased to 375 and 546, respectively, compared to early time points. In the SCA-susceptible SC1345 plants, unique upregulated DEGs increased from 6 hpi to 7 dpi (Fig. 3). The volcano plot based on RNA-seq data for each comparison is also presented as Figure S1.

### Temporal feeding by SCA differentiate genes unique to SCA-resistant and susceptible sorghum plants

**Fig. 4:**
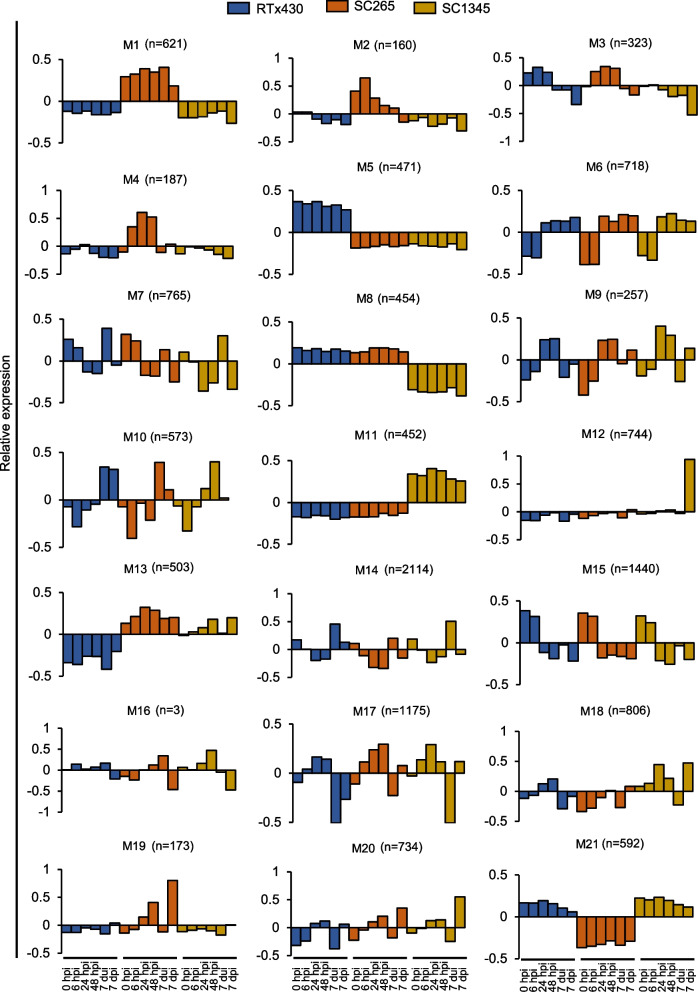
Weighted Gene Correlation Network Analysis (WGCNA) of differentially expressed genes in RTx430, SCA-resistant (SC265), and SCA-susceptible (SC1345) sorghum lines. M1 to M21 modules show expression patterns of a set of genes are assigned to the modules at 0, 6, 24, and 48 hpi, 7 dui and 7 dpi. hpi = hours post infestation, dui = days uninfested, and dpi = days post infestation, n = number of genes in each module

Weighted gene co-expression network analysis (WGCNA) was used to identify the genes sharing a common expression pattern among the six time points for the 13,266 DEGs. Twenty-one modules were generated, and each module showed the relative expression behavior of the genes grouped together (Fig. 4). Out of 21 modules (M), M1, M5, and M11 contained genes that displayed an elevated expression level in SC265, RTx430 and SC1345 plants respectively, at all the time points (Fig. 4). In contrast, M8, M13, and M21 had genes that were low expressed in SC1345, RTx430 and SC265 plants, respectively (Fig. 4). M2 genes show higher gene expression at 6 hpi in the SCA-resistant line, but their expression decreased over time from 24 hpi to 7 dpi. However, the genes of RTx430 and SC1345 plants had lower expression levels at all the time points in M2 (Fig. 4). M19 genes had higher expression levels in the SC265 plants after SCA infestation at both early and late time points (Fig. 4). M4 contained genes whose expression levels were high upon SCA infestation in the SCA-resistant SC265 plants only during the early time points (i.e., 6, 24 and 48 hpi), while the gene expression decreased at 7 dpi. However, the expression of these genes was lower in the uninfested conditions at 0 h and 7 day uninfested control plants compared to the infested conditions (Fig. 4). M6 genes expression level was lower at 0 and 6 hpi in all the lines but was higher at the remaining time points (Fig. 4). In contrast, the expression of genes in M15 was higher at uninfested (0 hpi) and 6 hpi but decreased at 24 and 48 hpi in all sorghum plants. The expression of genes of M15 at 7 day SCA-uninfested control plants remained low and did not change even after the aphid infestation in all sorghum plants (Fig. 4). M12 genes were downregulated across all the time points and lines, except for SC1345 at 7 dpi, which was upregulated. M7 module had genes with elevated expression levels in control condition and at 6 hpi only in all the sorghum lines. These modules were further used to categorize the genes expressed at early and late time points of SCA feeding.

### Gene enrichment analysis identifies several defense-related genes in the SCA-resistant line

**Fig. 5:**
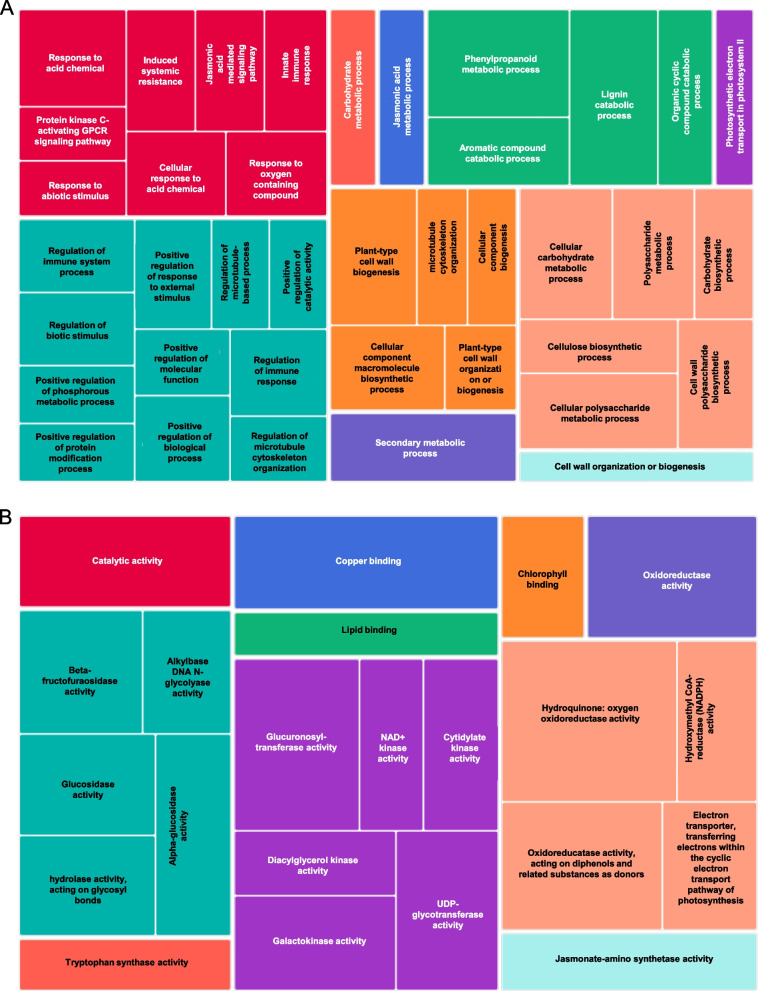
Gene ontology (GO) treemap of overrepresented GO terms in module 4 made by REVIGO program for **A**) Biological functions and **B**) Molecular functions. Each box represents the –log10 (*P*-value) of individual GO term and bigger size of the box reflects most significant GO terms. Similar functional categories with semantic similarity are represented in similar colored boxes

Gene enrichment analysis was performed to identify the functions of the genes comprised in a specific module. The biological processes and molecular functions corresponding to the genes were shown for the modules M4 (187 genes) and M19 (173 genes) (Fig. 5 and Figure S2). M4 contained genes that had higher expression in the SCA-resistant line at early time points but decreased expression in RTx430 and SCA-susceptible plants. The gene enrichment analysis showed that aphid feeding induced the genes involved in the biological processes such as phenylpropanoid metabolic process, JA-mediated signaling pathway, secondary metabolic process, immune response, induced systemic resistance, regulation of immune response, carbohydrate metabolism, cell wall organization and regulation of biotic stimulus (Fig. 5A and Figure S3A). Genes related to molecular functions such as oxidoreductase activity, hydroxyquinone-oxidoreductase activity, copper ion binding, sugar metabolism, jasmonate-amino synthetase activity, hydroxymethyl CoA-reductase (NADPH) activity, glucosidase activity and glucuronosyltransferase activity were also highly expressed in the SCA-resistant line (Fig. 5B and Figure S3B).

Module M19 contained genes whose expression were continuously increased from 6 hpi to 7 dpi with higher expression at 7 dpi in the SCA-resistant line. It constitutes genes related to biological processes such as carbohydrate metabolic process, vegetative to reproductive phase transition to meristem, development process involved in reproduction, cell wall synthesis, sucrose metabolic process, protein modification and ubiquitination, isoprenoid metabolic pathway, lipid metabolic process and photosynthesis (Figures S2A and S4A). Gene products modulating molecular functions were related to phospholipase D activity, glucosyltransferase activity, peptidase activity, UDP-glucosyltransferase activity, ATP dependent peptidase activity and phosphatidylethanolamine binding were found in this module (Figures S2B and S4B).

### Aphid feeding on SCA-resistant sorghum line increased the expression of genes related to cell wall formation and cuticular wax biosynthesis

To further explore the genes involved in plant defenses at early point of infestation, we investigated the genes that are part of M4 for their respective function (Supplemental Table S1). The first line of plant defense occurs through the interaction of herbivory with the plant cell wall. Aphid feeding on SCA-resistant sorghum line has induced the expression of cell wall-related genes. Three cellulose synthase genes (*SbiRTX430.03G320500*, *SbiRTX430.02G210900*, *SbiRTX430.01G231700*) were upregulated and had higher expression upon aphid feeding on resistant line, however their expression was lower on RTx430 and SCA-susceptible plants. Hydroxyproline-rich glycoproteins are important protein required for plant cell wall formation [40]. One gene (*SbiRTX430.02G429500*) was induced in RTx430, SCA-resistant and susceptible sorghum plants at 24 hpi, but the expression was relatively higher (FPKM_SC265_ = 612) in resistant line compared to the two other lines (FPKM_RTx430_ = 442 and FPKM_SC1345_ = 493). Another important cell wall forming protein is exostosin, which are glycoproteins in cell walls required for actin arrangements [41, 42]. Aphid feeding had induced the expression of two exostosin proteins (*SbiRTX430.09G170600* and *SbiRTX430.03G443400*) in the SCA-resistant line whereas this gene was downregulated in RTx430 and SCA-susceptible plants. *SbiRTX430.01G008800* (proline/lysine rich protein) is upregulated in the resistant line at 6 hpi, however, not differentially expressed in other two lines at any time points. Aphid infestation on SCA-resistant plants for 24 hpi upregulated the expression of cuticular wax biosynthesis-related gene, 3-ketoacyl-CoA synthase 6 (*SbiRTX430.03G191300*), which was not impacted by SCA feeding on RTx430 and SCA-susceptible plants.

### SCA feeding induced the upregulation of pathogenesis-related genes, ethylene and auxin signaling genes in the SCA-resistant sorghum line

Pathogenesis-related thaumatin (*SbiRTX430.02G284100*) was induced in the SCA-resistant line at 24, 48 hpi, and 7 dpi, whereas in RTx430, it was induced only at 7 dpi (Supplemental Table S1). Chitinases are also involved in affecting the insect herbivory for disrupting the cuticular membrane of insects [43]. Here, aphid herbivory induced the expression of chitinase gene (*SbiRTX430.01G545000*) in SCA-resistant line at 24 hpi (Supplemental Table S1). Three genes involved in ethylene biosynthesis (*SbiRTX430.02G226900*, *SbiRTX430.02G332100*, *SbiRTX430.04G301100*) were upregulated only in the SCA-resistant line, except for *SbiRTX430.02G226900*, which was also upregulated in the SCA-susceptible line at 24 and 48 hpi (Supplemental Table S1). Auxin-responsive *Gretchen Hagen3* (*GH3)* (*SbiRTX430.09G265900*), which is activated in stress conditions and also responds to auxin formation [44], was upregulated only in SCA-resistant SC265 genotype at 24 and 48 hpi (Supplemental Table S1).

### Prolonged SCA feeding on the SCA-resistant sorghum line induced the expression of genes related to proteinase inhibitors, sucrose metabolism, phospholipid metabolism and callose synthesis

To further identify the genes involved in defenses at later time points, we explored module M19 (Fig. 4). M19 contained 173 genes whose expression was continuously increased as the aphid feeding proceeded with the higher expression at 7 dpi (Supplemental Table S1). Proteinase inhibitors, which are important component of plant defense responses, accumulate via oxylipin pathway during the production of JA [45] and these compounds will interfere with insect digestive proteolytic activities. Here, SCA infestation had led to the increased expression of four protease/proteinase inhibitors at 7 dpi in the sorghum SCA-resistant line, such as cystatin B (*SbiRTX430.09G192300*), cysteine proteinase (*SbiRTX430.07G186000*), aspartic proteinase (*SbiRTX430.02G247500*) and serine carboxypeptidase-like 27 (*SbiRTX430.05G196500*) (Supplemental Table S1). Sucrose synthase responsible for sucrose metabolism in plants by converting sucrose to fructose and UDP glucose, which are further utilized in TCA cycle or other metabolic pathways [46]. Three sucrose synthase genes (*SbiRTX430.10G293900*, *SbiRTX430.04G376600*, *SbiRTX430.01G396400*) were upregulated in the SCA-resistant line at 7 dpi but not induced in the SCA-susceptible plants. Phospholipases activation lead to the production of defense signaling molecules such as oxylipins, JA and its derivatives and phosphatidic acid [47]. We found two phospholipases (*SbiRTX430.09G015400* and *SbiRTX430.09G062900*) upregulated in the SCA-resistant line at 7 dpi. Callose is a polysaccharide that is deposited at the site of insect feeding to restrict the insect stylet movement and pathogen progression in the plants. In our study, we found two callose synthase genes (*SbiRTX430.03G193100* and *SbiRTX430.03G192500*) that were upregulated in the resistant line at 7 dpi.

### Aphid feeding induced the expression of specific genes unique to resistant line at all the time points

Global transcriptomic analysis has shown that there were 21 DEGs (out of 13266 total DEGs identified) that were uniquely upregulated only in the SCA-resistant line after aphid infestation at all the time points (Supplemental Table S1). However, the expression of these genes was not induced in RTx430 and SCA-susceptible plants after SCA infestation. Protein kinases play significant role in activating the plant defenses in response to stress conditions [48]. Two protein kinase genes (*SbiRTX430.09G080300*, *SbiRTX430.07G228500*) were upregulated in the SCA-resistant line among the 21 DEGs. Expression of glycine and cysteine rich family protein genes (*SbiRTX430.02G022000*) was also induced at all time points. Glycine rich proteins are involved in regulating the responses to plant hormones such as SA, abscisic acid and ethylene and also modulate plant defenses [49]. Cysteine rich proteins are also part of defense-related compounds like antimicrobial peptides and plant defensins [50]. The expression of S-adenosyl-L-methionine-dependent *O*-methyltransferase (SAM-Mtases, *SbiRTX430.03G217400*) and *O*-methyltransferase 1 (*SbiRTX430.01G363800*), were higher in SCA-resistant line. SAM-Mtases are crucial enzymes in flavonoid and phenylpropanoid pathways, which are important defense-related pathways [51]. Plant defense-related genes such as NB-ARC gene (*SbiRTX430.05G178800*) and ethylene responsive transcription factor (*SbiRTX430.01G499200*) were also upregulated in the SCA-resistant line. Glycosyltransferases are responsible for transferring sugar moieties from UDP-activated sugar molecules to receptor molecules like lipids, secondary metabolites, and phytohormones [52]. Here, two genes (*SbiRTX430.10G272700* and *SbiRTX430.05G070400*), which are upregulated in response to aphid feeding, have their functions related to glycosyltransferase. Out of 21, there were six upregulated genes (*SbiRTX430.03G116200*, *SbiRTX430.09G176200*, *SbiRTX430.01G456800*, *SbiRTX430.03G348800*, *SbiRTX430.02G213500*, *SbiRTX430.03G086200*) whose functions are currently unknown.

### Transcriptional profile of genes involved in sorghum defenses

#### Defense-related genes

**Fig. 6:**
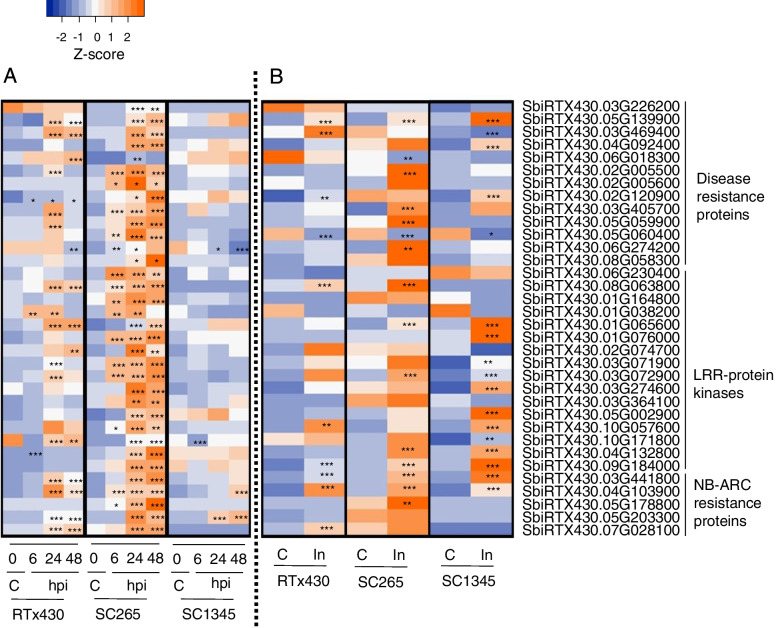
Heatmap of the relative expression level for the differential expressed genes (DEGs) in RTx430, SCA-resistant (SC265), and SCA-susceptible (SC1345) sorghum line. DEGs related to plant defenses at **A**) early time points after aphid infestation at 6, 24 and 48 hpi compared to control (0 hpi), and **B**) late time point after aphid infestation at day 7 (In; SCA-infested) compared to control (**C**; SCA-uninfested). Color scheme represents the normalized Z-score value. hpi = hours post infestation. Asterisks in the individual cell represents the significant difference as compared to the respective control for adjusted *P*-value (*** < 0.001, 0.001 < ** < 0.01, 0.01 < * < 0.05)

Genes related to plant defenses such as disease resistance responsive family proteins, LRR protein kinase family protein, NB-ARC domain-containing disease resistance protein were significantly upregulated in the SCA-resistant line (Fig. 6A). Thirteen disease resistance-related genes were upregulated at early time points (6, 24 or 48 hpi) of aphid infestation; however, all these genes were not differentially expressed in the SCA-susceptible line, except for *SbiRTX430.06G274200*, which was downregulated at 24 and 48 hpi (Fig. 6A). Similarly, 16 LRR-related genes were upregulated at early time points (6, 24, or 48 hpi) of aphid feeding and those genes were not differentially expressed in SCA-susceptible plants at all the time points. Only *SbiRTX430.10G171800* gene was downregulated in susceptible line at 6 hpi (Fig. 6A). The expression of five NB-ARC domain-containing genes was increased after aphid infestation at 6, 24 or 48 hpi. In the SCA-susceptible line, two out of five genes (*SbiRTX430.04G103900* and *SbiRTX430.05G203300*) were upregulated at 48 hpi, and 24 and 48 hpi respectively. At 7 dpi, only five disease resistance responsive family proteins (*SbiRTX430.05G139900*, *SbiRTX430.02G005500*, *SbiRTX430.03G405700*, *SbiRTX430.05G059900*, *SbiRTX430.06G274200*), five LRR protein kinase family proteins (*SbiRTX430.08G063800*, *SbiRTX430.01G065600*, *SbiRTX430.03G072900*, *SbiRTX430.04G132800*, *SbiRTX430.09G184000*), and three NB-ARC domain-containing disease resistance proteins (*SbiRTX430.03G441800*, *SbiRTX430.04G103900*, *SbiRTX430.05G178800*) were differentially upregulated in the SCA-resistant line (Fig. 6B). Five lipoxygenases (LOX) encoding 9-LOX (*SbiRTX430.01G129400, SbiRTX430.03G416000, SbiRTX430.03G416200, SbiRTX430.01G129600 and SbiRTX430.01G129500*) were upregulated upon SCA herbivory at early time points in SCA-resistant and RTx430 plants. *SbiRTX430.01G129400, SbiRTX430.03G416000* and *SbiRTX430.03G416200* were upregulated at all the time points in the SCA-resistant line (Supplemental Table S1).

#### JA biosynthesis

**Fig. 7:**
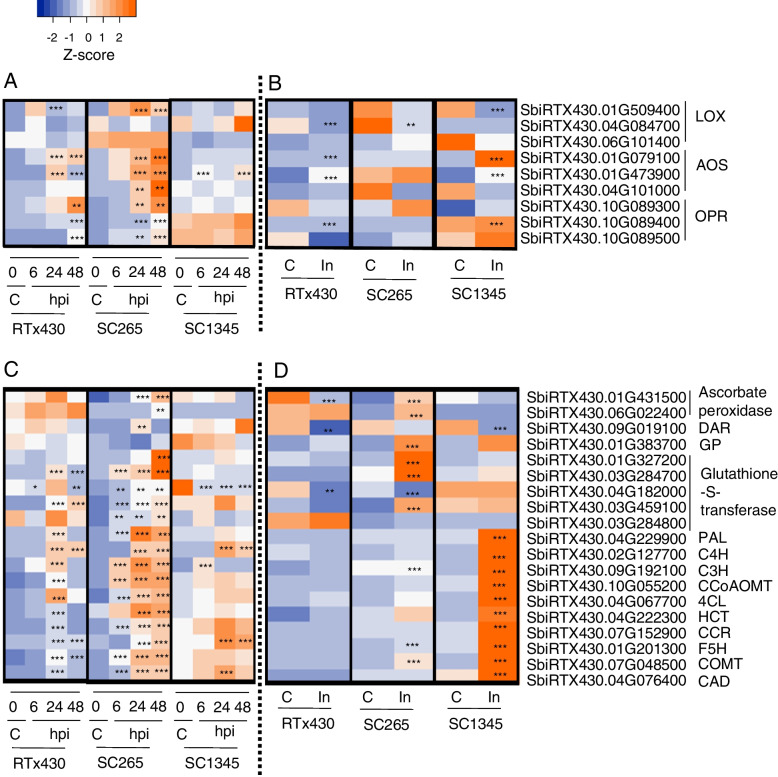
Heatmap of the relative expression level for the DEGs related to phytohormone, scavenging enzymes of reactive oxygen species (ROS), and monolignol biosynthesis pathway in RTx430, SCA-resistant (SC265), and SCA-susceptible (SC1345) sorghum lines. DEGs related to jasmonic acid biosynthesis at **A**) early time points after aphid infestation at 6, 24 and 48 hpi compared to control (0 hpi), and **B**) late time point after aphid infestation at day 7 (In; SCA-infested) compared to control (**C**; SCA-uninfested). DEGS related to ROS scavenging enzymes and monolignol biosynthesis pathway at **C**) early time points after aphid infestation at 6, 24 and 48 hpi compared to control (0 hpi), and **D**) late time point after aphid infestation at day 7 (In; SCA-infested) compared to control (C; SCA-uninfested). Color scheme represents the normalized Z-score value. LOX = 13-lipoxygenases, AOS = allene oxide synthase, OPR = 12-oxophyto-dienoate reductase 1, PAL = phenylalanine ammonia-lyase, C4H = cinnamate-4-hydroxylase, C3′H = p-coumaroyl quinate/shikimate 3′-hydroxylase, CCoAOMT1 = Caffeoyl-CoA O-methyltransferase, 4CL = 4-Coumarate:CoA ligase, HCT = p-hydroxycinnamoyltransferase, CCR = cinnamoyl CoA reductase 1, F5H = ferulate 5-hydroxylase 1, COMT = Caffeic acid O-methyltransferase 1, and CAD = Cinnamyl alcohol dehydrogenase. hpi = hours post infestation. Asterisks in the individual cell represents the significant difference as compared to the respective control for adjusted *P*-value (*** < 0.001, 0.001 < ** < 0.01, 0.01 < * < 0.05)

Biosynthesis of JA starts with the action of phospholipases in chloroplast membrane, which releases linolenic acid to chloroplast/plastid [53]. Lipoxygenase (LOX) enzymes act on linolenic acid to produce (13S)-hydroperoxyoctadecatrienoic acid, which is further catalyzed by allene oxide cyclase (AOC), allene oxide synthase (AOS), 12-oxophytodienoate reductase (OPR) to produce 12-OPDA [53]. Three 13-LOX genes annotation (*SbiRTX430.01G509400*, *SbiRTX430.04G084700* and *SbiRTX430.06G101400*) were identified and two of them (*SbiRTX430.01G509400* and *SbiRTX430.04G084700*) were induced upon SCA feeding at 24 and 48 hpi, and 7 dpi respectively (Fig. 7A and B). In RTx430 and SCA-susceptible SC1345 plants, *SbiRTX430.01G509400* was upregulated at 1 hpi and 7 dpi, respectively. In SCA-susceptible SC1345 plants, *SbiRTX430.01G129400*, *SbiRTX430.01G129500* and *SbiRTX430.01G129600* were induced only at 6, 24, and 48 hpi respectively, but were not induced at any other time points. *SbiRTX430.03G416200* and *SbiRTX430.01G509400* were not induced in the SCA- susceptible plants. Aphid feeding also induced three genes encoding AOS (*SbiRTX430.01G079100*, *SbiRTX430.01G473900* and *SbiRTX430.04G101000*) in the SCA-resistant line at early time points. Two of these genes were not impacted by SCA feeding in SCA-susceptible plants at early time points but *SbiRTX430.01G473900* was upregulated at 6 and 48 hpi. Three genes related to OPR (*SbiRTX430.10G089400*, *SbiRTX430.10G089300*, *SbiRTX430.10G089500*) were also upregulated at different early time points (24 and 48 hpi) in the SCA-resistant line but not induced in susceptible line except for *SbiRTX430.10G089400,* which was upregulated at 7 dpi (Fig. 7A and B).

#### Reactive oxygen species

Reactive oxygen species (ROS) are known to play important roles in plant growth and development and also protect the plants from biotic and abiotic stresses [54]. Insect attack on plants leads to the production of highly reactive forms of oxygen like hydrogen peroxide, superoxide, and hydroxyl radical [55]. In the current study, genes related to scavenging enzymes such as ascorbate peroxidase (*SbiRTX430.01G431500* and *SbiRTX430.06G022400*), dehydroascorbate reductase (*SbiRTX430.09G019100*), glutathione peroxidase (*SbiRTX430.01G383700*), glutathione-S-transferase (GSTs) (*SbiRTX430.01G327200*, *SbiRTX430.03G284700*, *SbiRTX430.04G182000*, *SbiRTX430.03G459100* and *SbiRTX430.03G284800*), which detoxify the excess ROS, were upregulated in the SCA-resistant line at early time points (6, 24, and 48 hpi) (Fig. 7C). Six genes were upregulated at 7 dpi as well in the SCA-resistant line (Fig. 7D). However, these genes were either mostly downregulated or not differentially expressed in RTx430 and SCA-susceptible SC1345 plants.

#### Monolignol biosynthesis pathway

Lignin is a plant biopolymer present in the secondary cell wall and provides mechanical support to the plants. Lignin also protects plants against environmental stresses by acting as a barrier [56]. The abundance of lignin can alter the penetration of insect stylet to feed on phloem cells. In our study, gene enrichment analysis shows that the aphid infestation triggers the lignin metabolism in the SCA-resistant line at early time points. Ten genes involved in monolignol pathways were induced upon aphid infestation in all three lines, namely *SbiRTX430.04G229900* (*PAL*, phenylalanine ammonia-lyase 2), *SbiRTX430.02G127700* (*C4H*, cinnamate-4-hydroxylase), *SbiRTX430.09G192100* (*C3H*, 4-coumarate hydroxylase), *SbiRTX430.10G055200* (*CCoAOMT1*, caffeoyl-CoA O-methyltransferase), *SbiRTX430.04G067700* (4CL, 4-coumarate:CoA ligase 2), *SbiRTX430.04G222300* (*HCT*, hydroxycinnamoyl transferase), *SbiRTX430.07G152900* (*CCR*, cinnamoyl CoA reductase 1), *SbiRTX430.01G201300* (*F5H*, ferulic acid 5-hydroxylase 1), *SbiRTX430.07G048500* (*COMT*, caffeic acid O-methyltransferase 1), and *SbiRTX430.04G076400* (*CAD*, cinnamyl alcohol dehydrogenase). All the ten genes were upregulated at early time points (i.e., 6, 24, and 48 hpi) in the SCA-resistant line except for *C4H*, *HCT* and *F5H*, which were not differentially expressed at 6 hpi (Fig. 7C). *CCoAOMT1*, *F5H* and *COMT* were also upregulated at 7 dpi (Fig. 7D). In contrast to the SCA-resistant line, these ten genes were upregulated at 24 hpi and 7 dpi in the RTx430 and SCA-susceptible plants, respectively (Supplemental Table S1). In RTx430, *C4H*, *F5H*, and *COMT* were also upregulated at 48 hpi and in the SCA-susceptible line, *C4H*, and *F5H* were also upregulated 24 and 48 hpi (Fig. 7C). Besides these 10 genes, *SbiRTX430.06G157300*, *SbiRTX430.07G078300* and *SbiRTX430.06G015900* encoding CAD were also upregulated at 1 and 7 dpi in the SCA-resistant line, which aligns with our previously published proteomic study on SC265 upon SCA feeding at 1 and 7 dpi [57] (discussed below).

### Comparative analysis of transcriptomics with proteomic study

Previously, proteomic analysis of SCA-resistant sorghum SC265 genotype infested with aphids at 1 and 7 dpi was performed and 158 differentially expressed proteins (DEPs) were identified in response to aphid feeding [57]. Further, we have shown that feeding by SCA suppressed the plant defenses at early time points (1 dpi) by downregulating the DEPs related to signal transduction, cell wall, and secondary metabolism [57]. Interestingly, at 7 dpi, plants were able to overcome the aphid attack by inducing DEPs related to pathogenesis-related proteins, protease inhibitors and oxidative stress signaling [57]. Here, we performed the comparison of transcriptomic data for 1 and 7 days with the previously conducted proteomic analysis to understand how the genes expressing at transcriptomic level undergoes into translation [57] (Table 1 and Supplemental Table S1). Among all the compared DEPs with DEGs, we found that at 1 dpi, 54 DEGs were upregulated whereas only 16 DEPs were upregulated at the at proteomic level (Table 1) and were related to photosynthesis and plant growth. At 1 dpi, only three common DEPs and DEGs were upregulated (Table 2). Similarly, the upregulated DEGs and DEPs at 7 dpi were 51 and 50, respectively. Among these 38 were the commonly upregulated ones, and those DEPs and DEGs were regulating the oxylipins, pathogenesis-related proteins, secondary metabolism, oxidative metabolism and stress signaling. There were 10 DEGs and 49 DEPs that were downregulated at 1 dpi. We also identified the DEGs that were having contrasting patterns at the proteome level. For example, 34 DEGs that were upregulated at the transcript level were downregulated at the proteomic level (Table 2). After prolonged aphid feeding, the number of DEGs and DEPs downregulated were 13 and 10, respectively (Table 1).

**Table 1:** Number of differentially expressed genes (DEGs) and differentially expressed proteins (DEPs) upregulated or downregulated in SC265 plants after aphid feeding for 1 and 7 days in a comparative study

Days post infestation (dpi) by aphids	Sorghum line (SC265)		
	Differentially expressed	# of genes (DEGs)	# of proteins (DEPs)
1 dpi	Upregulated	54	16
	Downregulated	10	49
7 dpi	Upregulated	51	50
	Downregulated	13	10

**Table 2:**
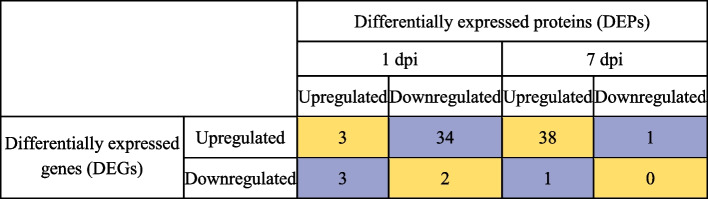
Comparison of the number of differentially expressed genes (DEGs) and differentially expressed proteins (DEPs) in SC265 plants after aphid feeding for 1 and 7 days for similarities and dissimilarities status of DEGs and DEPs. Similarities are represented in rows with orange color and dissimilarities are represented in rows with blue color

*dpi* Days post infestation

## Discussion

The current study illustrates the sorghum transcriptomic responses to the aphid herbivory on RTx430, SCA-resistant (SC265) and SCA-susceptible (SC1345) plants compared at the early (6, 24 and 48 hpi) and late (7 dpi) time points of SCA attack. The results have shown that SCA-resistant sorghum line initiates the early defenses within 6 hpi by rapidly inducing disease resistance proteins (R-proteins), ROS, JA signaling and monolignol pathway, whereas the later defenses were related to activation of sucrose metabolism, phospholipid metabolism and callose synthase genes and proteinase inhibitors. The study has provided insights into the underlying genetic mechanism of resistance in sorghum against SCA and shown that resistant plants are responding swiftly and mount defenses at early times of aphid herbivory. On the other hand, SCA-susceptible plants lack the ability to rapidly induce early defenses.

Previously, it was shown that *Arabidopsis* and rice plants that were infested with green peach aphids and rice stem borer (*Chilo suppressalis*), respectively, had higher number of upregulated DEGs compared to downregulated DEGs [58, 59], suggesting that the insect feeding triggers the reorganization of the host transcriptome. Similarly, our DEG analyses have shown that number of upregulated genes in the SCA-resistant line was higher compared to SCA-susceptible and RTx430 plants, which also aligns with a previous study where SCA feeding induced higher percentage of upregulated genes compared to downregulated genes in the SCA-resistant sorghum line [31]. Gene enrichment analysis differentiated the plant responses to short and prolonged aphid feeding in the SCA-resistant line. At early time points, aphid feeding induced the expression of genes corresponding to secondary metabolic process, jasmonate signaling, induced systemic resistance, redox activity, cell wall organization and carbohydrate metabolism (Fig. 4A and Figure S2A). Additionally, GO terms related to lignin and phenylpropanoid metabolic and catabolic process were highly enriched. Further screening of the genes related to these pathways confirmed their involvement in plant defenses (discussed later).

Oxidoreductases are predicted to be involved in sorghum defense against SCA [31]. Our results also suggest that oxidoreductase activity was highly enriched in the SCA-resistant plants after aphid feeding. Interestingly, oxidoreductases have been identified in the saliva of *M. persicae* and *Bemisia tabaci* [60], suggesting that these insects can potentially use oxidoreductases for detoxifying the plant defense compounds. Sugars act as a critical energy resource in plants, however, alterations in sugar levels may provide toxicity to insects. Previously, we found that SCA feeding on sorghum JA-deficient plants altered the sugar metabolism and enhanced the sugar levels compared with wild-type plants. Moreover, sugars when incorporated into the aphid diet resulted in decreased aphid fecundity compared with SCA reared on diet alone, suggesting that sugars may have direct negative impact on SCA proliferation [15]. In the current study, we found three sucrose synthase genes that were upregulated in the SCA-resistant line after aphid feeding. Our data also showed upregulation of genes at 7 dpi related to protease/proteinase inhibitors like cystatin B, cysteine proteinase, aspartic proteinase and serine carboxypeptidase-like 27. These data further confirms our proteomic data of proteinase inhibitors, which were also induced at 7 dpi at the proteome level [57].

Plants display multitude of defenses to survive from insect attack by activating downstream signaling pathways upon recognition of herbivore-associated molecular patterns. For example, recognition of insect salivary elicitors by the R-proteins (NBS-LRR) trigger immunity in plants. *Mi1.2* gene of tomato, a NBS-LRR gene confers resistance to potato aphid (*M. euphorbiae*) [61], root-knot nematode (*Meloidogyne* spp.) [62] tomato psyllid (*Bactericerca cockerelli*) and sweet potato whitefly (*B. tabaci*) [63]. Similarly, in a recent study on SCA-sorghum transcriptomics, several NBS-LRR genes were identified and suggested that these genes may be responsible for conferring sorghum resistance to SCA. Our study also identified several NBS-LRR genes, which were uniquely induced in the SCA-resistant sorghum line, whereas they were not induced in RTx430 and SCA-susceptible plants after SCA attack. Collectively, these data indicate a possible role of NBS-LRR defense-related proteins in mediating sorghum resistance to SCA.

Several studies have shown that JA and ethylene are involved in plant defense signaling during stress conditions [29, 64]. As mentioned before, we have previously shown that JA has a dual role in providing resistance and susceptibility at different stages of aphid colonization [15]. In the current study, we found eight JA biosynthesis genes that were highly expressed in the SCA-resistant line upon aphid feeding at early time points. However, at later time point only one gene in the 13-LOX pathway was upregulated. Similar JA patterns at the metabolome and transcriptome provide evidence that JA induction at early time points after SCA attack (i.e., up to 48 hpi) may contribute to sorghum defense against SCA [15, 57]. Similarly, 12-oxo-phytodienoic acid (OPDA), which is a precursor of JA, is also known to provide maize (*Zea mays*) resistance to corn leaf aphid [65]. Similar to a previous study on SCA-sorghum interactions [32], our study also revealed elevated expression of ethylene biosynthesis genes in the SCA-resistance line, suggesting the critical role of ethylene in sorghum defense against SCA. Heightened levels of auxin in plants may cause susceptibility to invading pathogens. In citrus, *GH3*, which is an auxin early response gene, functions to reduce the levels of free auxin and to enhance the defense responses [44]. In our study, we also identified one *GH3* gene that was upregulated in the SCA-resistant line at 24 and 48 hpi. However, the precise role of *GH3* in maintaining auxin levels after SCA attack in sorghum plants is yet to be determined.

ROS play a key role in protecting plants from different environmental stresses and activation of ROS further triggers the downstream defense signaling [66]. However, increased ROS accumulation can be toxic for plants if not quenched in a timely manner [66]. Different detoxifying enzymes such as catalases, superoxide dismutase, glutathione peroxidases, glutathione S transferases and ascorbate peroxidases are involved in maintaining the levels of ROS. In soybean (*Glycine max* (L.), infestation by cowpea aphid (*Aphis craccivora*) increased the production of H_2_O_2_. However, peroxidases were able to regulate the levels of H_2_O_2_ through the antioxidant activity [67]. Peroxidases also contributed to defense against cowpea aphid by being anti-nutritive and toxic. In the current study, we found nine ROS scavenging genes, which were specifically upregulated in the SCA-resistant sorghum line at early time points of SCA infestation. This signifies that the plant is rapidly initiating the production of ROS to mount the defenses and scavenging enzymes are acting on ROS to maintain the homeostasis. Our results align with the recent findings in sorghum where SCA feeding evoked the expression of GSTs providing defense to sorghum plants [5]. Additionally, ROS signaling induces the production of the SA, which further regulates the expression of PR proteins. Upregulation *PR*, a SA marker gene, in the SCA-resistant line after SCA feeding (i.e., 24, 48 hpi) indicates that ROS and SA signaling may occur simultaneously. Taken together, our data suggests that the defense response in the SCA-resistant line be potentially displayed through the production of ROS.

Lignin is a major constituent of plant cell wall and is involved in plant defenses against pest, pathogens, or wounding [58, 59, 68]. Besides lignin, many secondary metabolites such as phenols and flavonoids are also produced through the phenylpropanoid pathway [58]. In our current study, gene enrichment analysis shows that the aphid infestation triggers the phenylpropanoid and lignin metabolism in the SCA-resistant line at early time points. Aphid feeding induced 10 monolignol pathway genes at early time points in the SCA-resistant line. We also observed similar results on transcriptomic and proteomics data comparison, in which PAL and CAD were differentially upregulated at the transcript and protein levels at 7 dpi in the SCA-resistant line [57]. Overexpression of *MYB* transcription factors increased the production of lignin and reduced the proliferation of aphids on chrysanthemum [69, 70]. Lignin accumulation may contribute to thickening cell walls, thereby restricting aphid feeding. Contrastingly, genes associated with monolignols and phenols were suppressed during fall armyworm (FAW; *Spodoptera frugiperda*) feeding on FAW-resistant sorghum line, suggesting that plants may divert its resources to other secondary metabolites, for example, flavonoids [58]. However, we observed a reverse trend in the current study where SCA feeding induced the expression of genes associated with monolignols in the SCA-resistant line. It is plausible that the different modes of feeding behaviors displayed by aphids and caterpillars and their associated cues may lead to the activation of distinct sorghum defense responses.

## Conclusions

In this study, we utilized NAM founder lines SC265 and SC1345, which are SCA-resistant and susceptible lines, respectively, to understand the mechanism of plant defenses in sorghum against SCA feeding. We found that plants respond rapidly to effectively initiate its defense signaling via production of ROS, JA signaling, monolignol pathway and activation of disease resistance proteins. The current comprehensive understanding of how sorghum plants responding to SCA can contribute to the knowledge of plant defenses. The availability of recombinant inbred lines generated from SC265 and SC1345 lines with reference line RTx430 can provide the opportunity to further evaluate the QTLs corresponding to the differentially expressed genes in response to SCA feeding. Additionally, in our transcriptome study, we identified ~ 22 percent of total DEGs whose functions are not yet annotated, and some of those genes were highly expressed in the SCA-resistant line. It is plausible that their functions may contain information on plant defenses and may turn out as a useful resource for pest management strategies. Finally, the use of resistant sorghum can help to reduce the dependence on chemicals and provides a durable solution for insect management.

## Methods

### Plant and insect materials

The three sorghum nested association mapping population (NAM) founder lines used in this study were obtained from the United States Department of Agriculture-Germplasm Resources Information Network (USDA-GRIN) global germplasm, USA, and were grown and propagated at the University of Nebraska-Lincoln (UNL) greenhouse facilities. The USDA-GRIN provides these seed/plant genetic resources freely available to educational institutions and were grown at UNL permitted spaces. The lines we used were: i) RTx430 is the elite reference line, ii) SC265 is the SCA-resistant line and iii) SC1345 is the SCA-susceptible line. For aphid feeding assays, seeds were sown in commercial potting mix (PRO-MIX BX BIO FUNGICIDE + MYCORRHIZAE, Premier Tech Horticulture Ltd., Canada) in conical pots. Plants were raised in a 16/8 h light/dark photoperiod, watered daily, and fertilized at intervals of 4 days (N:P:K::20:10:20). Plants at the three-leaf stage were used to conduct experiments. The SCA colony was started from a single parthenogenic female aphid collected from aphid-infested sorghum plants at Louisiana State Agricultural Center Dean Lee Research Station, Alexandria, LA [32]. The SCA colony was maintained on the highly susceptible BCK60 sorghum in the conditions described above. New plants were introduced to the colony at weekly intervals to generate a continuous supply of aphids.

### Plant tissue collection for transcriptomics study

Treatments included SCA-infested and uninfested (control) plants for the three sorghum lines, RTx430, SC265 and SC1345. Times for sample collection included four early time points (0, 6, 24, and 48 h post infestation [hpi]) and one late time point (7 days post infestation [dpi] and 7 days SCA-uninfested control plants). Plants were infested with 10 adult aphids for treatment while uninfested plants served as control treatments. The aphids were placed on second leaf opposite to the whorl with the help of a fine brush and then enclosed in clip cages. The clip cages were removed after two days to provide enough area for aphids to reproduce and the plants were covered with cylindrical cages. At each specified time points, leaf tissue was cleared from aphids, and leaf samples from three plants were pooled to form one biological replicate, and three replicates per time point were used for each line. Leaf tissue samples were harvested in 2 mL vials containing stainless steel beads. The samples were immediately flash-frozen in liquid nitrogen and stored at -80 °C.

### RNA-Seq library construction

Leaf tissue was homogenized in a 2010 Geno/Grinder (SPEX SamplePrep) for 60 s in presence of liquid nitrogen to prevent thawing of the tissue. Total RNA was extracted using Zymo Research RNA Clean & Concentrator (Research Irvine, CA, USA) following the manufacturer protocol. RNA quantity was measured by Nanodrop 2000c Spectrophotometer (Thermo Scientific, Waltham, MA, USA). Two micrograms of RNA per time point were submitted to Novogene for stranded mRNA-seq library preparation and Illumina RNA sequencing. Sequencing of mRNA-seq libraries generated 150 bp paired-end with 20 million reads on average per library. Raw data are available in SRA under the bioproject PRJNA716317 (https://dataview.ncbi.nlm.nih.gov/object/PRJNA716317?reviewer=2clh3m19tqmc89c6seo1t49nfv).

### RNA-Seq analysis

RNA-Seq libraries were assessed for a quality check using FASTQC [71]. Trimming of reads was done using Trimmomatic v0.39 [72]. The reads with Phred score less than 20 and read length less than 45 bp were removed. Filtered reads were mapped to sorghum reference genome RTx430 v2.1 (https://phytozome-next.jgi.doe.gov/) using Tophat2 [73]. Parameters for mapping include 0 splicing mismatch (-m 0) and 0 mismatches (-N 0) for the samples collected from the line RTx430 and 1 mismatch (-N 1) for SC265 and SC1345. Transcript reconstruction was made using Cufflinks v2.2.1 and parameters included frag-bias-correct (-b), multi-read-correct (-u) and quantification against the reference annotation only (-G) (Supplemental Table S2). DEG analysis was generated using Cuffdiff 2.2.1 and DEGs were found and described as the significantly expressed genes with *q*-value < 0.05 and fold-change|log_2_(FPKM_Infested_/FPKM_Contol_)|≥ log_2_(2) [74] as compared with control: 0 h vs 6 hpi, 0 h vs 24 hpi, 0 h vs 48 hpi; and for later time points: SCA-uninfested (Control) vs SCA-infested (7 dpi). Gene co-expression network was performed using R packages i) WGCNA to create co-expression modules and identify the sets of DEGs that express in a similar pattern across lines and time points [75] and ii) stats [76]. The gene ontology enrichment analysis was performed using GOBU [77]. The *p*-values were computed using Fisher’s exact test with module *P*-value and multiple testing was done to obtain corrected *p*-values using the R module called ‘*P*-adjust’. The GO terms with *P* ≤ 0.05 were further used and analyzed through REVIGO to produce non-redundant GO terms (http://revigo.irb.hr/) represented in Treemap [78]. Raw FPKM values of differentially expressed genes were transformed to Z-score with the formula z score = (FPKM-Avg.)/SD, where FPKM is individual gene value at each condition, Avg. is the mean FPKM of each gene at all conditions and SD is the standard deviation. Heatmaps were prepared using gplots package with heatmap.2 function in R studio. Blue color represents low gene expression and orange color represents high gene expression. KEGG (Kyoto Encyclopedia of Genes and Genomes) [79, 80] pathway were obtained with the Plant GeneSet Enrichment Analysis Toolkit (http://structuralbiology.cau.edu.cn/PlantGSEA/analysis.php) [81] by using the sorghum gene ID geneID.Btx623.v3.1.1 (Supplemental Table S1) and the parameters: statistical method: Fisher, multi-test adjustment method: Bonferonni and significance level 0.05. Volcano plots were created using the R package ggplot2.

### Supplementary Information

**Additional file 1: Supplemental Table 1.** Gene expression contrast analysis.

**Additional file 2: Supplemental Table 2.** Mapping statistics.

**Additional file 3: Supplemental Figure 1.** Volcano plots based on the RNA-seq data for each comparison. Red indicates a significant differential expression (adjusted *P*-value<0.05 and |log2(FC)| >=1). **Supplemental Figure 2.** Gene ontology (GO) treemap of overrepresented GO terms in module 19 made by REVIGO program for A) Biological functions and B) Molecular functions. **Supplemental Figure 3.** Gene enrichment analysis of differentially expressed genes (DEGs) in module M4. **Supplemental Figure 4.** Gene enrichment analysis of differentially expressed genes (DEGs) in module M19.

## Data Availability

The raw datasets generated during the sequencing of current study are available in SRA under the bioproject PRJNA716317 (https://www.ncbi.nlm.nih.gov/bioproject/PRJNA716317).

## Abbreviations

DEGs: Differentially Expressed Genes
DEPs: Differentially Expressed Proteins
DPI: Days Post Infestation
FDR: False Discovery Rate
GO: Gene Ontology
HPI: Hours Post Infestation
JA: Jasmonic acid
LOX: Lipoxygenase
SA: Salicylic acid
SCA: Sugarcane aphid
FPKM: Fragments Per Kilobase Million
ROS: Reactive Oxygen Species
