# Supplementary material for: Temporal transcriptomic profiling elucidates sorghum defense mechanisms against sugarcane aphids
Source: BMC Genomics. 2023 Aug 5;24:441. doi: 10.1186/s12864-023-09529-5 (PMC10403856; doi:10.1186/s12864-023-09529-5)
Supplement: Supplementary file 3 — Additional file 3: Supplemental Figure 1. Volcano plots based on the RNA-seq data for each comparison. Red indicates a significant differential expression (adjusted P-value<0.05 and |log2(FC)| >=1). Supplemental Figure 2. Gene ontology (GO) treemap of overrepresented GO terms in module 19 made by REVIGO program for A) Biological functions and B) Molecular functions. Supplemental Figure 3. Gene enrichment analysis of differentially expressed genes (DEGs) in module M4. Supplemental Figure 4. Gene enrichment analysis of differentially expressed genes (DEGs) in module M19. [file 12864_2023_9529_MOESM3_ESM.pdf]

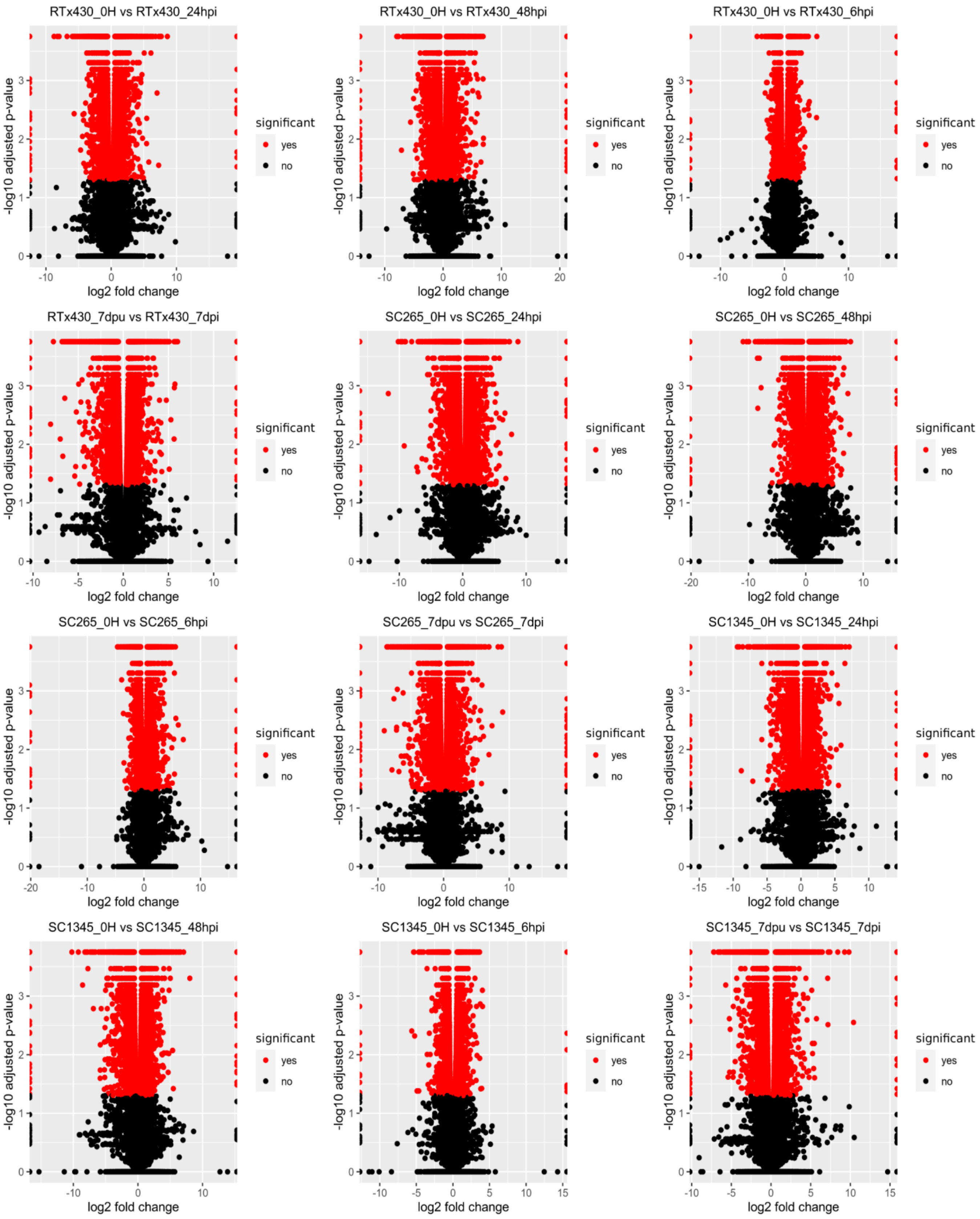

Figure S1. Volcano plots based on the RNA-seq data for each comparison. Red indicates a significant differential expression (adjusted *P*-value<0.05 and |log2(FC)| >=1).

Supplemental Figure 2
Puri et al.

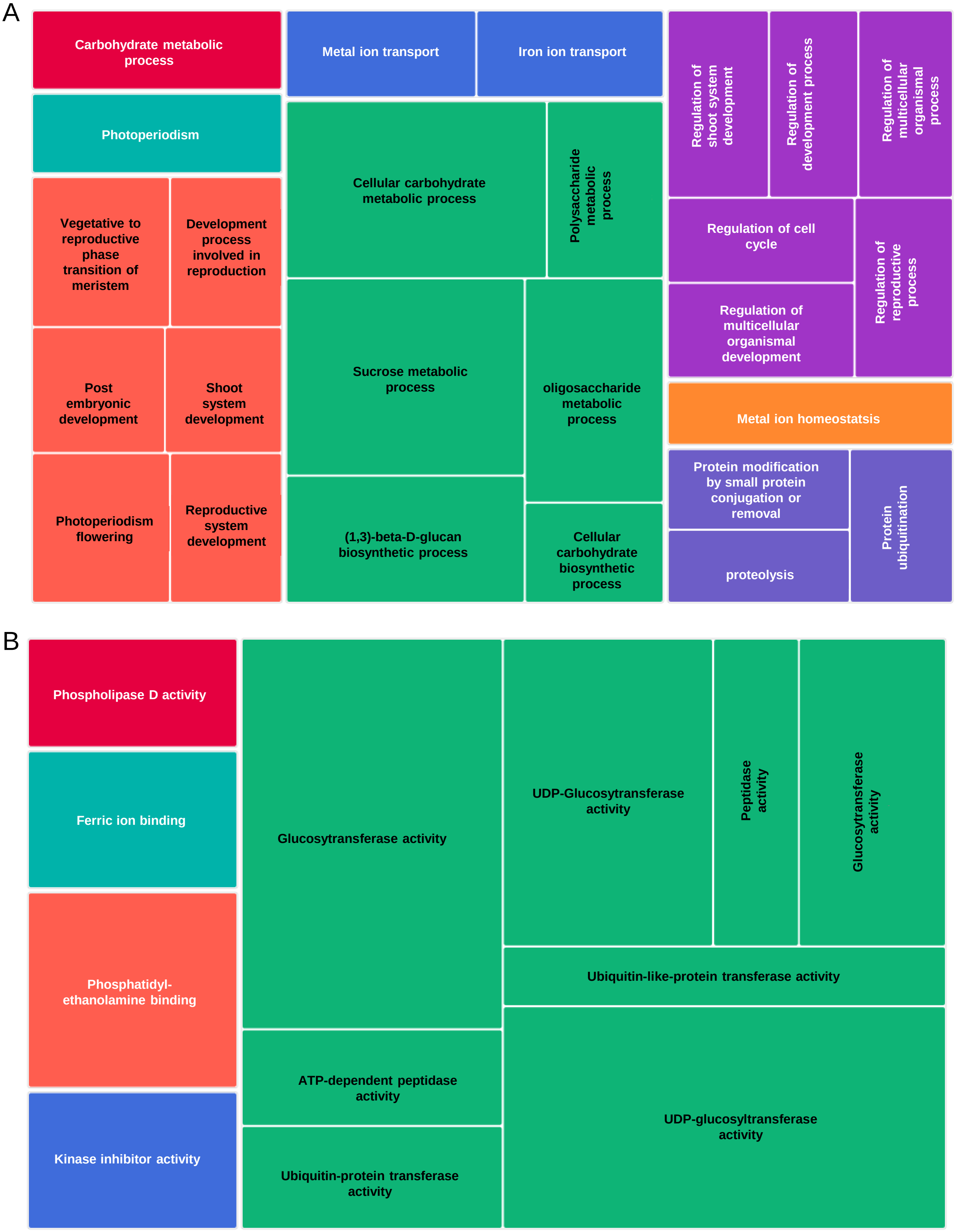

Figure S2. Gene ontology (GO) treemap of overrepresented GO terms in module 19 made by REVIGO program for A) Biological functions and B) Molecular functions. Each box represents the –log10 (*P*-value) of individual GO term and bigger size of the box reflects most significant GO terms. Similar functional categories with semantic similarity are represented in similar colored boxes.

Supplemental Figure 3
Puri et al.

A

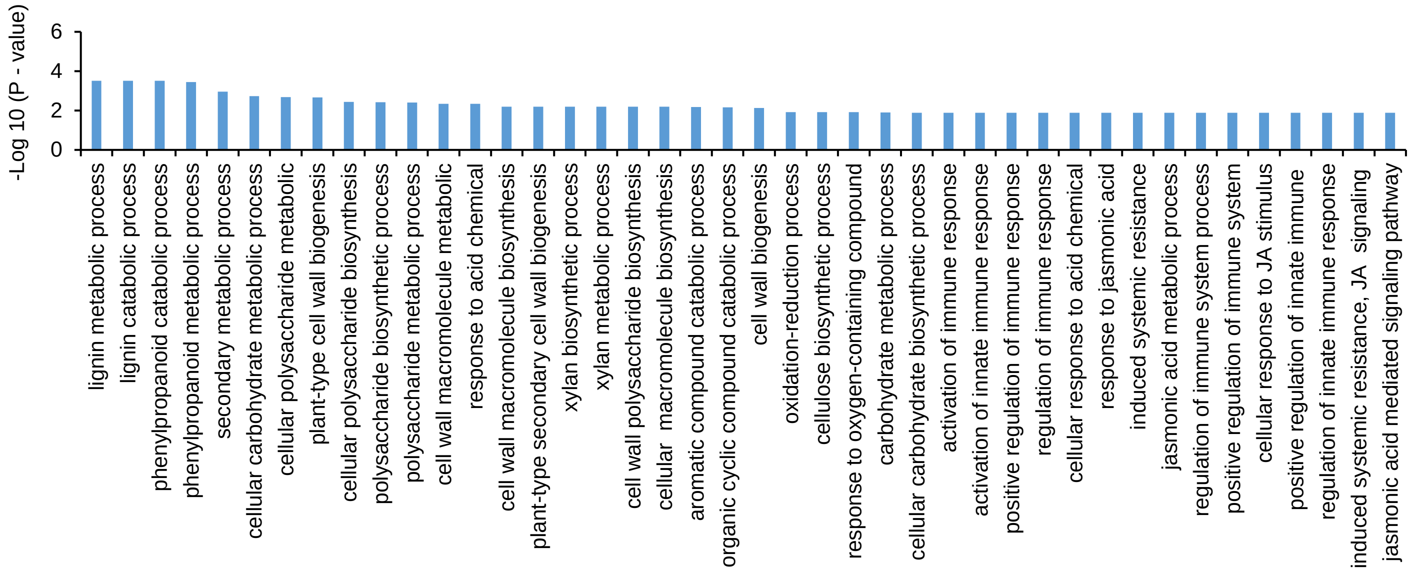

B

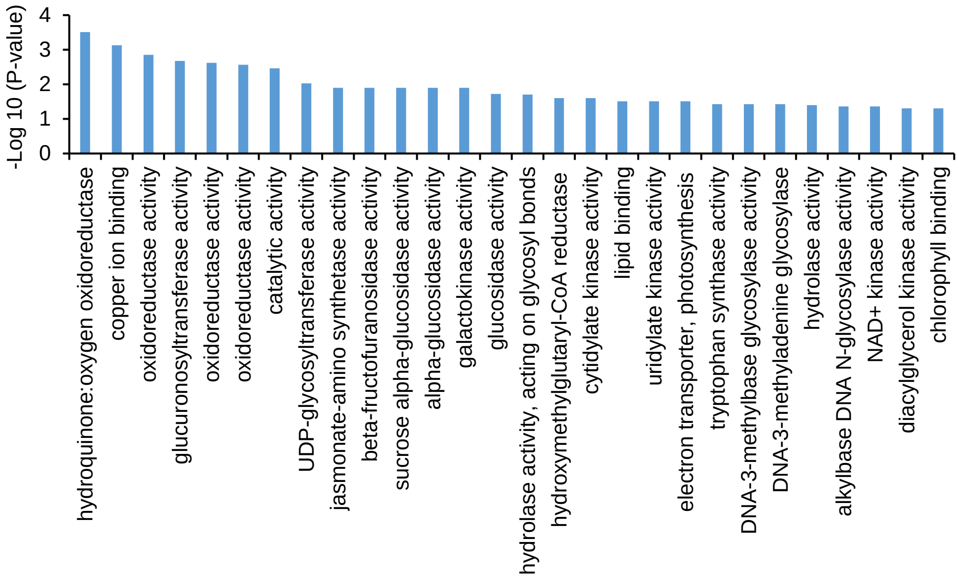

Figure S3. Gene enrichment analysis of differentially expressed genes (DEGs) in module M4. Significantly enriched genes of A) Biological functions and B) Molecular functions are plotted against the negative logarithm of *P*-value ($P<0.05$).

Supplemental Figure 4
Puri et al.

A

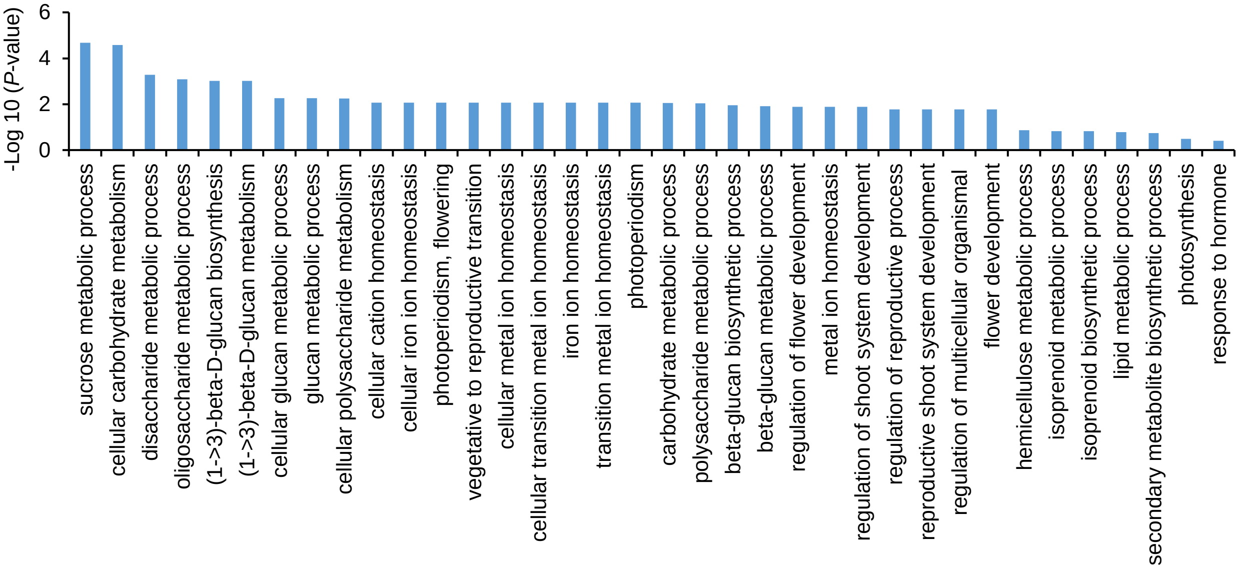

B

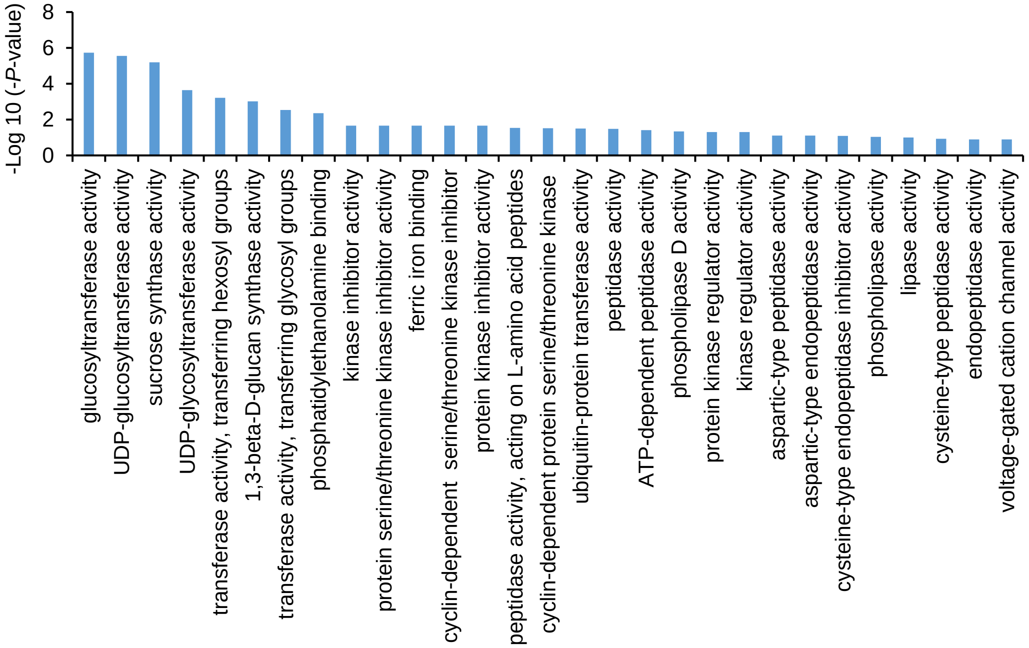

Figure S4. Gene enrichment analysis of differentially expressed genes (DEGs) in module M19. Significantly enriched genes of A) Biological functions and B) Molecular functions are plotted against the negative logarithm of *P*-value (*P*<0.05).
